# Theranostic Platforms Based on Silica and Magnetic Nanoparticles Containing Quinacrine, Chitosan, Fluorophores, and Quantum Dots

**DOI:** 10.3390/ijms23020932

**Published:** 2022-01-15

**Authors:** Dmitry V. Korolev, Galina A. Shulmeyster, Natalia V. Evreinova, Maria S. Syrovatkina, Maria S. Istomina, Victor N. Postnov, Ilia V. Aleksandrov, Aleksandr S. Krasichkov, Michael M. Galagudza

**Affiliations:** 1 Institute of Experimental Medicine, Almazov National Medical Research Centre, 2 Akkuratova Str., 197341 Saint Petersburg, Russia; korolev_dv@almazovcentre.ru (D.V.K.); g.schulmeister@yandex.ru (G.A.S.); zna47@lti-gti.ru (N.V.E.); ist_mary@mail.ru (M.S.I.); postnovvn@mail.ru (V.N.P.); galagudza_mm@almazovcentre.ru (M.M.G.); 2 Laboratory of Biophysics of Blood Circulation, Pavlov First Saint Petersburg State Medical University, 6–8 L’va Tolstogo Street, 197022 Saint Petersburg, Russia; 3 Department of Electrochemical Production, St. Petersburg State Technological Institute Technical University, 26 Moskovsky pr., 198003 Saint Petersburg, Russia; 4 Department of Micro- and Nanoelectronics, Saint Petersburg Electrotechnical University “LETI”, 5 Professora Popova Street, 197376 Saint Petersburg, Russia; mashachuro@yandex.ru; 5 Institute of Chemistry, Saint Petersburg State University, 7/9 Universitetskaya Emb., 199034 Saint Petersburg, Russia; 6 Department of Radio Engineering Systems, Saint Petersburg Electrotechnical University “LETI”, 5 Professora Popova Street, 197376 Saint Petersburg, Russia; krass33@mail.ru; 7 Department of Pathophysiology with Clinical Pathophysiology Course, Pavlov First Saint Petersburg State Medical University, 6–8 L’va Tolstogo Street, 197022 Saint Petersburg, Russia

**Keywords:** theranostics, multilayer nanoparticles, magnetic nanoparticles, silica nanoparticles, chitosan, quinacrine, indocyanine green, colloidal quantum dots

## Abstract

In this paper, we describe the synthesis of multilayer nanoparticles as a platform for the diagnosis and treatment of ischemic injuries. The platform is based on magnetite (MNP) and silica (SNP) nanoparticles, while quinacrine is used as an anti-ischemic agent. The synthesis includes the surface modification of nanoparticles with (3-glycidyloxypropyl)trimethoxysilane (GPMS), the immobilization of quinacrine, and the formation of a chitosan coating, which is used to fix the fluorophore indocyanine green (ICG) and colloidal quantum dots AgInS_2_/ZnS (CQDs), which serve as secondary radiation sources. The potential theranostic platform was studied in laboratory animals.

## 1. Introduction

Chitosan is considered to be a promising biodegradable polymer and is used in various fields of biomedicine. In particular, it is used for targeted drug delivery [1,2,3] and also as the basis of systems developed for the controlled release of drugs [4]. Researchers are interested in the possibility of using chitosan nanoparticles (CNPs) modified with fluorescent dyes, such as indocyanine green, fluorescein, or colloidal quantum dots [5,6], as labels for in vitro and in vivo imaging. The process is characterized by the increased time required for the excretion of such fluorescent labels from the body, which is an important factor for in vivo diagnostics.

Chitosan can ensure the controlled release of a drug from certain organs and lesions. The authors of [7] described the synthesis of Fe_3_O_4_@C/carboxymethyl cellulose (CMC)/chitosan composite microparticles. Fe_3_O_4_ nanoparticles were carbonized via thermal treatment of polyethylene glycol, and then the nanoparticles (NPs) were incorporated into the CMC matrix and coated with chitosan by a self-assembly method to form core–shell polyelectrolyte complexes. The particles included diclofenac sodium with loading of up to 70%, which is released in a pH range of 6.8–7.4, mainly in the stomach area. In [8], zinc oxide nanoparticles were embedded in a CMC matrix and then coated with chitosan using a self-assembly method to form core–shell polyelectrolyte complexes. This platform was loaded with the anticancer drug 5-fluorouracil. Similar to the previous one, this platform also showed pH-sensitive properties and released the active substance in the stomach at a reduced pH value.

In the field of sorbents, significant research has been focused on inorganic nanoparticles coated with chitosan. The high content of amino groups in chitosan determines its effective use as a sorbent, as well as the possibility of chemical modifications. For instance, in [9], the authors developed chitosan-coated Al_2_O_3_/Fe_3_O_4_ nanoparticles intended for use as an effective adsorbent for cationic azo dye removal. In [10], spherical magnetic calcium-modified microparticles were modified with chitosan using crosslinking and then used as an effective adsorbent for the removal of orange II dye. In [11], the authors described nanoparticles intended for the removal of heavy metal ions Hg^2+^, Pb^2+^, and Cu^2+^. Composite Fe_3_O_4_@SiO_2_@ nanoparticles were obtained by synthesizing the magnetite core and coating it with silicon oxide, followed by the crosslinking of chitosan with glutaraldehyde. The authors reported that the composite nanoparticles showed a nearly monodisperse distribution of 105 nm sized particles with a core diameter of 80 nm and a chitosan layer thickness of 12 nm. The magnetic Fe_3_O_4_@Zr-CTS microspheres described in [12] were obtained by coating magnetite particles with chitosan through a coordination reaction between zirconium oxychloride and chitosan. This adsorbent has been used to remove hexavalent chromium (VI) from aqueous solutions. In [13], the researchers described the synthesis of Fe_3_O_4_ nanoparticles coated with polyaniline. In order to fix the polyaniline, magnetite nanoparticles were modified with chitosan. The obtained nanoparticles had a spherical core–shell structure with a uniform size of about 100 nm and a 20–30 nm diameter core. Microspheres have been used as magnetic adsorbents for the extraction of aromatic compounds. Multifunctional magnetic iron oxide nanoparticles have been used for cancer theranostics [14]. Hybrid organic/inorganic nanoparticle nanotechnology has also been used in cancer theranostics [15].

An interesting trend has been the synthesis of chitosan microspheres for drug delivery systems. Chitosan microspheres are obtained by the emulsion crosslinking method using various crosslinking agents [16,17] to deliver the adsorbed drug and prolong the potential duration of its action. An interesting and more technologically advanced extension of this approach is the synthesis of multi-liposomal containers [18] by electrostatic adsorption of anionic liposomes on the surface of cationic chitosan nanoparticles. Such agglomerates break down into particles of 10–15 nm in the presence of proteolytic enzymes and release substances encapsulated in liposomes. In [19], the authors proposed obtaining an effective sorption material by modifying carbon fiber with chitosan by ionic gelation using sulfate ions.

Sometimes, chitosan is modified to produce a drug or a delivery vector. In [20], chitosan-polynitroxides were studied as an antioxidant agent that effectively reduced the level of reactive oxygen species. Mannosylated chitosan has been proposed for the delivery of antituberculosis drugs to macrophages [21].

The use of chitosan as a coating material seems to be very promising, since each fragment of this biopolymer has an active amino group. However, currently, there are only a small number of published studies on the use of chitosan as a coating material for drug immobilization. Most of the studies have focused on sorption.

The purpose of the present study was to synthesize multilayer silica and magnetite-based nanoparticles modified with chitosan as a platform intended for theranostic applications. The particles contain fluorophore dyes, quantum dots, and the drug quinacrine and, as shown in [22,23], and have a positive effect on postischemic myocardium recovery.

## 2. Results and Discussion

### 2.1. Synthesis Concept and Development

In this study, we designed a theranostic platform for the diagnosis and treatment of ischemic injuries. In this design, either SNPs or MNPs were used as the carrier, and quinacrine was used as the anti-ischemic agent [22,23]. ICG and CQDs were used as a source of secondary radiation.

**Figure 1:**
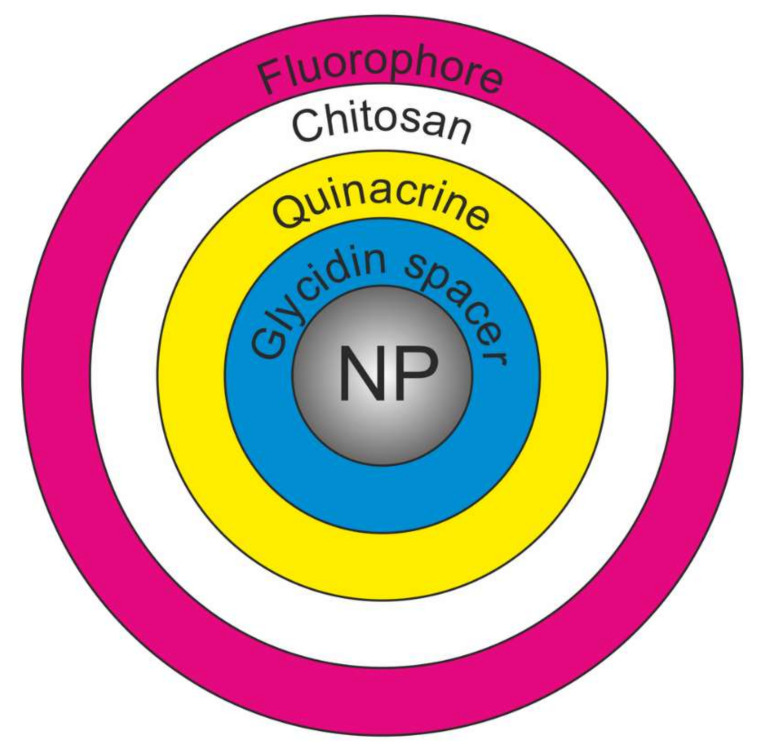
Schematic diagram of a core–shell nanostructure, SNP(MNP)-GPMS-quinacrine-chitosan-fluorophore.

First, a glycidine spacer was grafted onto the SNP surface. Then, quinacrine was covalently immobilized. Next, chitosan was applied to the preparation, and a secondary radiation source (e.g., ICG or CQDs) was immobilized in it. A schematic diagram of this core–shell nanostructure is shown in Figure 1.

**Figure 2:**
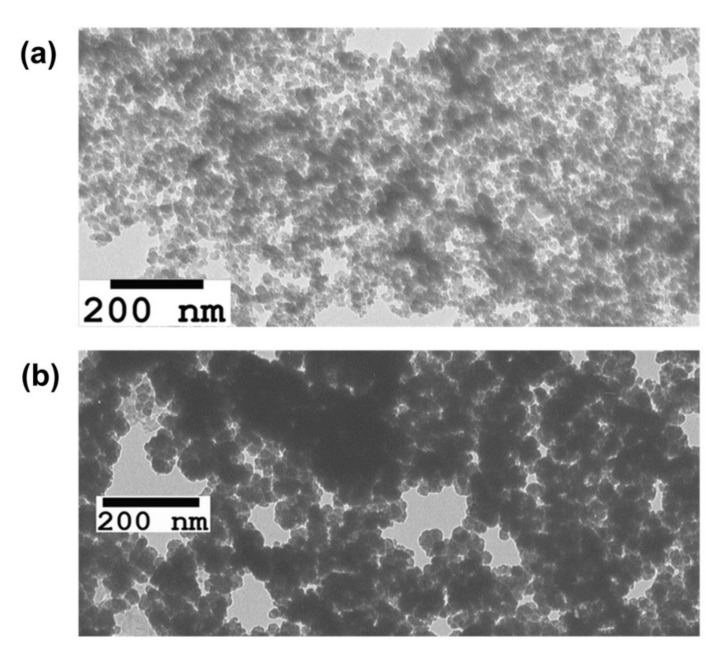
TEM images of precursor nanomaterials: (**a**) SNP; (**b**) MNP.

The aerosol particle size was 10–15 nm, the magnetite particle size was 40–50 nm (Figure 2), and the specific surface areas were 200 m^2^/g and 100 m^2^/g, respectively. A large specific surface area enables a high (200 m^2^/g) drug capacity, and the small size of the particles makes it possible to successfully overcome the reticuloendothelial system. It is worth mentioning that such carriers have been used quite widely in nanomedicine [24]. The presence of hydroxyl groups on the surface provides evidence for the potential application of carriers in the creation of complex grafted surface compounds, including platforms intended for theranostic applications. In the present study, we present a technique developed for modifying the surface of magnetite and aerosol nanoparticles, which involves the immobilization of the drug quinacrine, creation of a chitosan coating, and fixation of the fluorophore indocyanine green and quantum dots.

**Figure 3:**
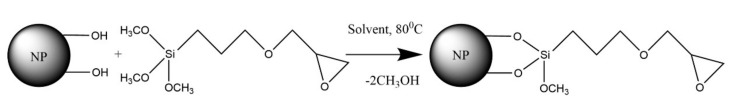
Glycidyl spacer synthesis diagram.

For covalent immobilization of quinacrine, the surfaces of nanoparticles were modified with (3-glycidyloxypropyl)trimethoxysilane according to the diagram shown in Figure 3.

This modifier was chosen due to the high reactivity of its epoxy groups and because of the possibility for further modifications. However, it should be noted that the concentration of grafted groups when using this reagent is usually significantly lower than in the case of the chemisorption of aminoalkoxysilanes due to the catalytic effect of amines on the interaction of silica with this reagent [25]. In the case of GPMS chemosorption, the addition of amines, which are often used as catalysts for the chemical modification of a silica surface with chloro- and alkoxysilanes, is excluded by the chemical properties of the epoxy groups. In this regard, several studies have been conducted to determine the optimal solvent for the liquid-phase modification of magnetite and aerosol with GPMS. Benzene, toluene, and cyclohexane have been used as solvents.

As can be seen from the obtained data (Table 1), aromatic solvents yield values close to those of grafted group concentrations per unit of surface area for magnetite particles. In the case of silica, this value is much lower. The highest concentration of the modifier is observed on magnetic nanoparticles with cyclohexane used as a solvent. This value is significantly higher than the maximum grafting density observed when the silica surface is modified by aminoalkoxysilanes (4.0 µmol/m^2^) [25] and exceeds the hydroxyl group concentration on the extremely hydroxylated surface (7.6 µmol/m^2^) [26]. Therefore, it is reasonable to argue that the use of cyclohexane on a magnetite surface does not result in the formation of a glycidine spacer monolayer; however, it results in the formation of a polymer layer. Because the solvents used in the experiments were dried, it can be assumed that coordinated water, a small amount of which might be found on the surface after freeze drying, promoted GPTM polycondensation and the formation of a polymer layer. A more significant influence of this factor on surface modification is apparent when using nonpolar cyclohexane; in this case, water generally does not enter the solution and interacts with the modifier on the surface. The IR spectra of the GPMS-modified sample showed absorption bands that may be regarded as epoxy group vibrations in the range of 1240–1260 cm^−1^, whereas symmetric stretching vibrations of the ring of the molecule occurred in the range of 950–860 cm^−1^, and antisymmetric stretching vibrations of the ring occurred in the range of 865–750 cm^−1^ [27,28].

**Figure 4:**
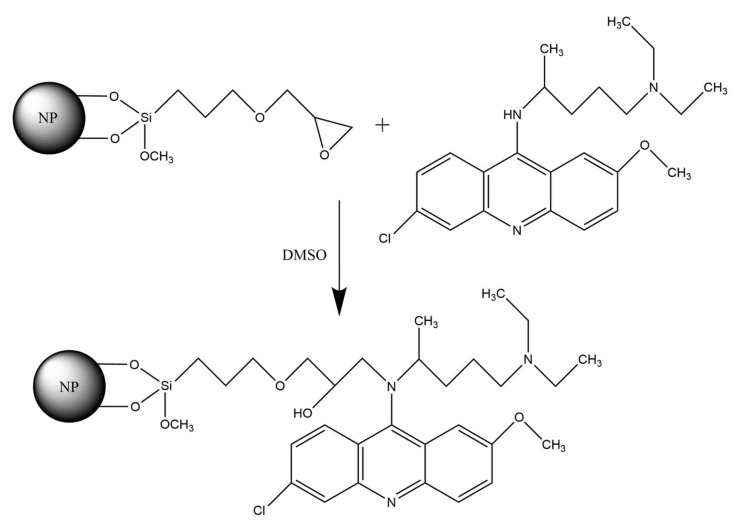
Quinacrine immobilization diagram.

Glycidine spacers were used to immobilize quinacrine according to the schematic diagram shown in Figure 4.

The data on the amount of immobilized quinacrine are presented in Table 2. By comparing the quinacrine content and the glycidine spacer concentration (Table 1), it can be concluded that there is no correlation between these values. This may be due to the abovementioned influence of water on the GPMS chemisorption, forming a dense polymer film, in which only accessible epoxy groups located on its surface can interact with quinacrine.

**Figure 5:**
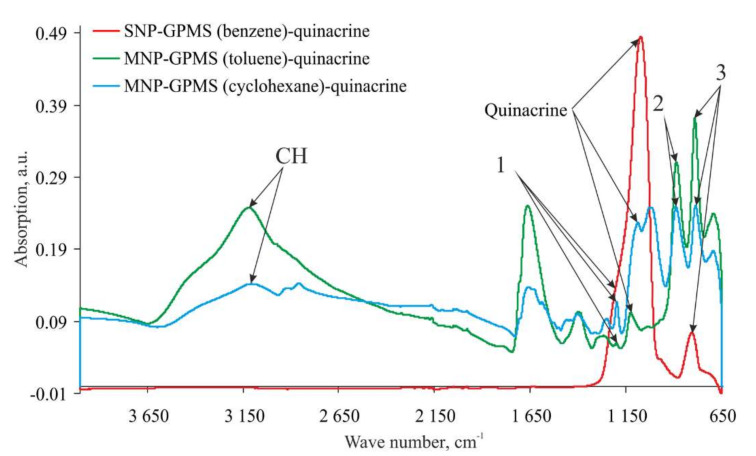
IR spectra of the GPMS-modified nanoparticles with immobilized quinacrine.

The IR spectra of GPMS-modified nanoparticles with immobilized quinacrine are shown in Figure 5. The spectra show that all three samples contain glycidine ring residues: bands 1, 2, and 3 for samples on magnetic nanoparticles and band 3 on silica nanoparticles. This can be explained by the fact that instead of a GPMS monolayer, a rather dense polymer shell consisting of spacer molecules is formed around the nanoparticle. Here, quinacrine only interacts with GPMS molecules located on the shell surface. In the sample with silica nanoparticles, bands 1 and 2 are not present. This is probably due to the thinner shell and, as a consequence, to the more complete GPMS reaction with quinacrine.

All three samples show a strong band corresponding to the wavenumber 1130 cm^−1^. This band is not present in the reference spectrum of the GPMS-modified magnetic nanoparticles. Therefore, it can be attributed to the immobilized quinacrine. Since quinacrine was covalently immobilized, a 1230 cm^−1^ shift of the reference band was possible, which was observed in the samples based on magnetic and silica nanoparticles.

**Figure 6:**
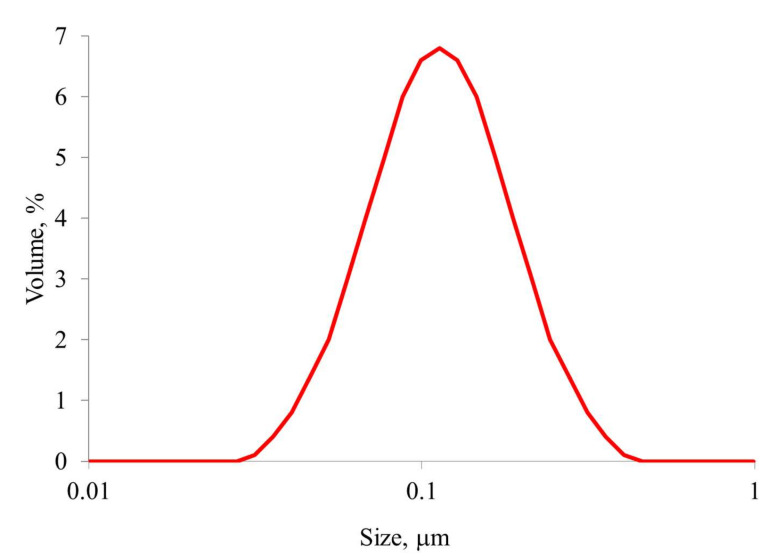
Particle size distribution of SNPs with quinacrine immobilized on a glycidyl spacer.

The average size of SNPs with quinacrine immobilized on a glycidyl spacer was 100 nm (Figure 6). It should be noted that particles of this size are used in nanomedicine [29].

In vitro studies performed using fluorescence tomography have shown that quinacrine-modified nanoparticles have sufficient luminous efficiency for in vivo imaging and can be used for theranostic applications [30]. The results are presented in Table 3.

Then, chitosan was applied to the surface of the obtained nanoparticles in order to introduce ICG or CQDs, and the optimal acid reagents necessary for modification with chitosan were determined.

The efficiency of the application of chitosan was investigated by determining the ICG immobilization capacity (Table 4). The analysis was carried out on SNPs, since MNP particles have a partial fluorescence quenching effect.

A chitosan coating was not formed from the oxalic acid solution under any investigated conditions. The coating obtained from the chitosan solution in citric acid appeared to be the most effective; perhaps, in this case, the acid acted as a crosslinking agent.

**Figure 7:**
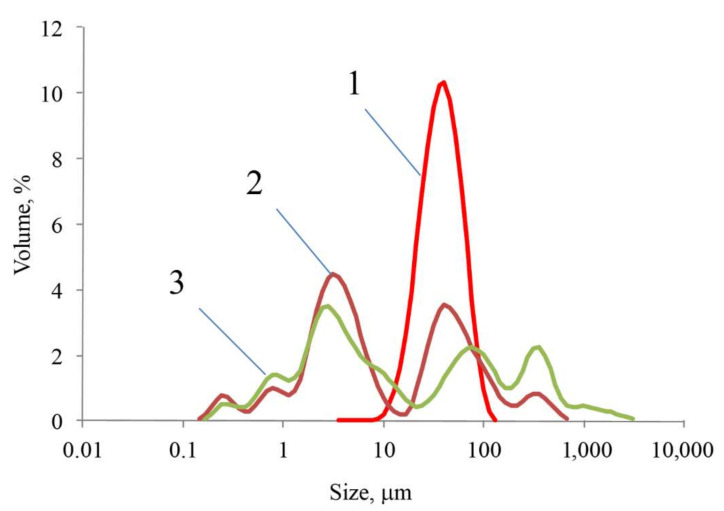
Particle size distribution upon chitosan condensation from acid solutions: (1) acetic acid; (2) oxalic acid; (3) citric acid.

Chitosan condensation from acid solutions produced rather large particles (Figure 7). In acetic acid, the average diameter of the particles was about 50 μm; in oxalic and citric acids, particle sizes ranged from 1 to 600 microns.

**Figure 8:**
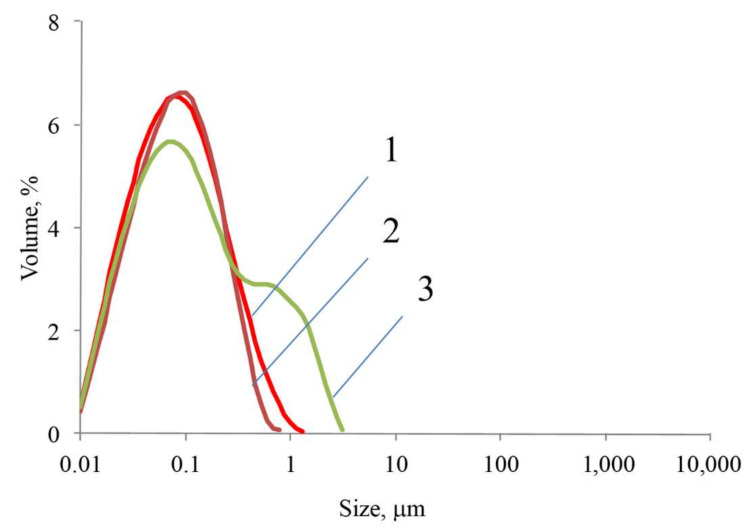
Particle size distribution upon chitosan condensation on SNPs from acid solutions: (1) acetic acid; (2) oxalic acid; (3) citric acid.

The introduction of SNP nucleation centers led to a sharp decrease in the average particle diameter (Figure 8). Particles formed in acetic and oxalic acid solutions were similar in size. The average particle diameter upon chitosan condensation on SNP and upon the immobilization of carboxylated quantum dots was 100 nm, while upon ICG immobilization, it was about 70 nm.

ICG or CQDs were immobilized on the chitosan coating. In vitro optical fluorescence of these samples was measured on a fluorescence tomograph; the measurement results are shown in Table 5.

Chitosan forms a porous coating on the nanoparticle surface, which is supported by the finding that the amount of fluorophore immobilized on the chitosan coating is much higher than the value predicted to be immobilized on a spherical particle of this size. In addition, the authors of [31,32] also showed that unmodified and modified glycol chitosan coatings both form pores. In this case, crosslinking from different acids probably produces different porosity and pore size values. Therefore, ICG molecules can penetrate the pores where the ICG fluorescence is compromised by the shell material. This may explain the high level of fluorescence shown by the acetic acid sample. It is possible that the chitosan coating in this sample is less porous than the samples prepared with the use of other acids.

### 2.2. Investigation of Physical and Chemical Properties

**Figure 9:**
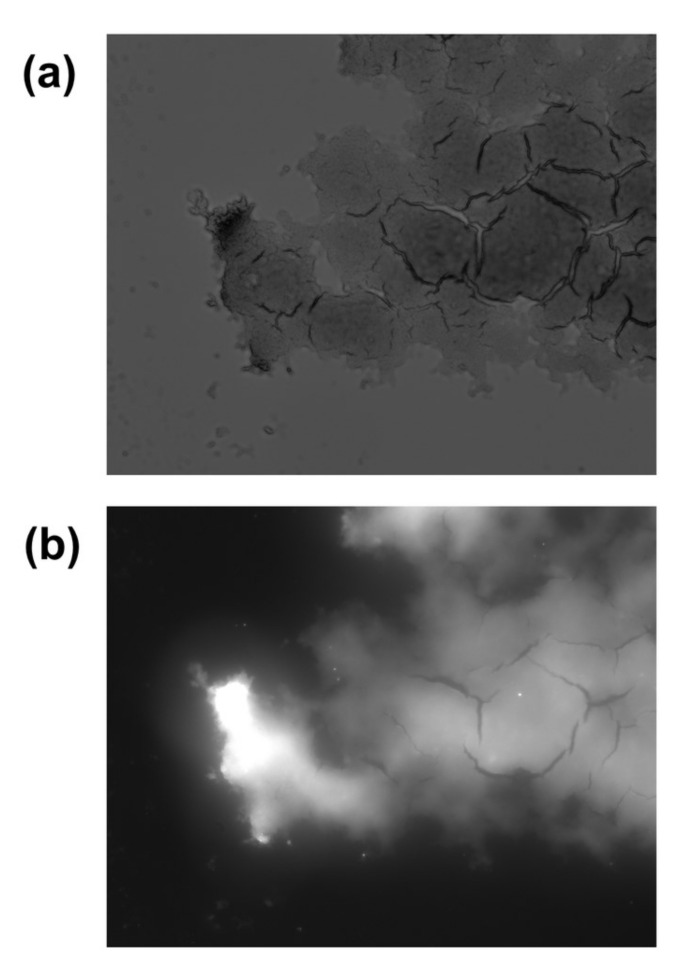
The study of conjugate using a fluorescent microscope: (**a**) light channel; (**b**) channel with quinacrine fluorescence cutoff.

The use of a fluorescence microscope (Figure 9) made it possible to prove the presence of quinacrine in the nanocomplex under a layer of chitosan and indocyanine green. The image, isolated using light filters for the quinacrine emission wavelength, demonstrates clear fluorescence upon excitation with ultraviolet light (Figure 9b) as compared with the light channel (without light filters) (Figure 9a).

Data obtained by measurement using an Ivis Lumina LT fluorescence tomograph are presented in Table 6. The samples were analyzed for fluorescence of quinacrine, indocyanine green, and colloidal quantum dots AgInS_2_/ZnS.

The level of fluorescence of all obtained samples was sufficient for visualization on a fluorescence tomograph. The highest luminous efficiency was shown by a sample with a secondary CQD shell and with the emitted radiation filter for quinacrine. This is due to the fact that a part of the CQD radiation overlaps with the quinacrine secondary radiation area. The result was obtained as the sum of the intensities.

**Figure 10:**
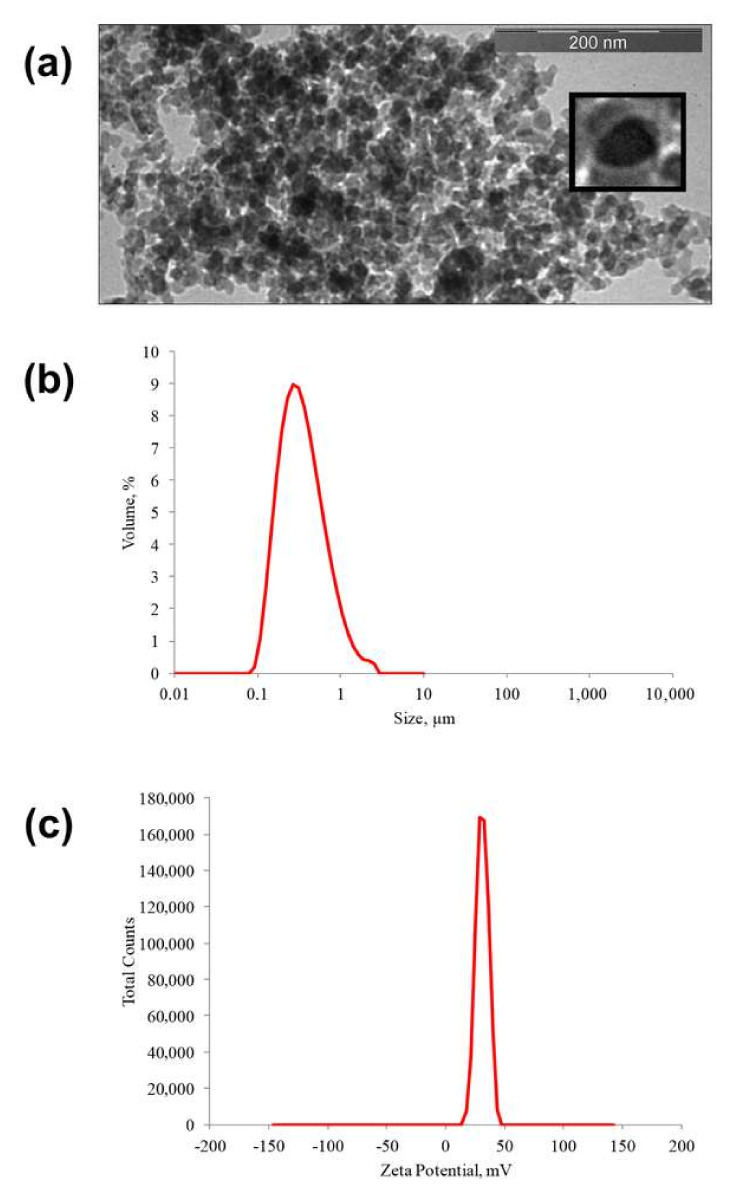
Physical properties of the obtained nanoobject “core–shell” structure: (**a**) TEM photograph of the obtained multilayer nanostructures; (**b**) distribution of the hydrodynamic diameter; (**c**) zeta potential.

As can be seen from Figure 10a, multilayer nanostructures SNP-GPMS-quinacrine-chitosan-ICG, for the most part, have a globular shape. The size of nanoparticles mainly ranges from 20 to 40 nm. These nanoparticles can be classified as “core–shell”-type particles. The dark-colored particle nuclei obviously contain silica, while the light shells contain a layer of organic modifiers. In an aqueous solution, such particles form associations about 200 nm in size (Figure 10b) and have a zeta potential of about 30 mV (Figure 10c).

### 2.3. Research in Laboratory Animals

**Figure 11:**
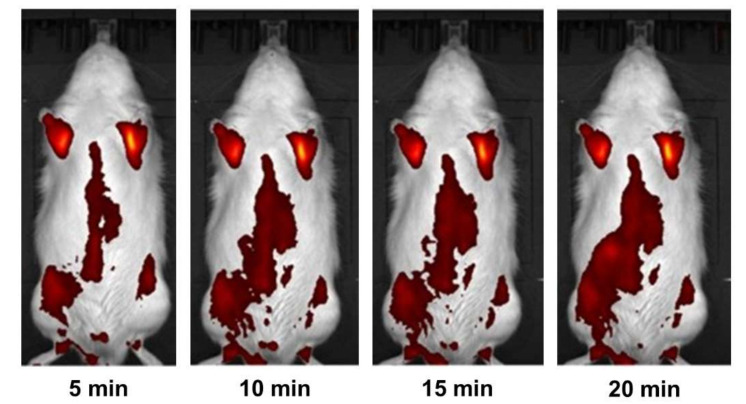
Time study of intravenous drug administration using fluorescence tomography (background, BKG subtracted).

The study of in vivo samples by fluorescence tomography with a secondary shell of indocyanine green was carried out on male SPF Wistar rats that were previously anesthetized with isoflurane by intravenous administration of 2 mL of the drug with a concentration of 2 mg/mL. The result is shown in Figure 11. The distribution of the drug was observed for 20 min. The figure shows that the drug spread throughout the animal’s body and had begun to be metabolized in the liver by 20 min.

The study on the effect of passive targeted delivery of the multilayer structure was based on the myocardial ischemia–reperfusion model and carried out on male SPF Wistar rats. At the end of the experiment, the animals were euthanized, and the organs were harvested and sliced for examination by fluorescence tomography.

**Figure 12:**
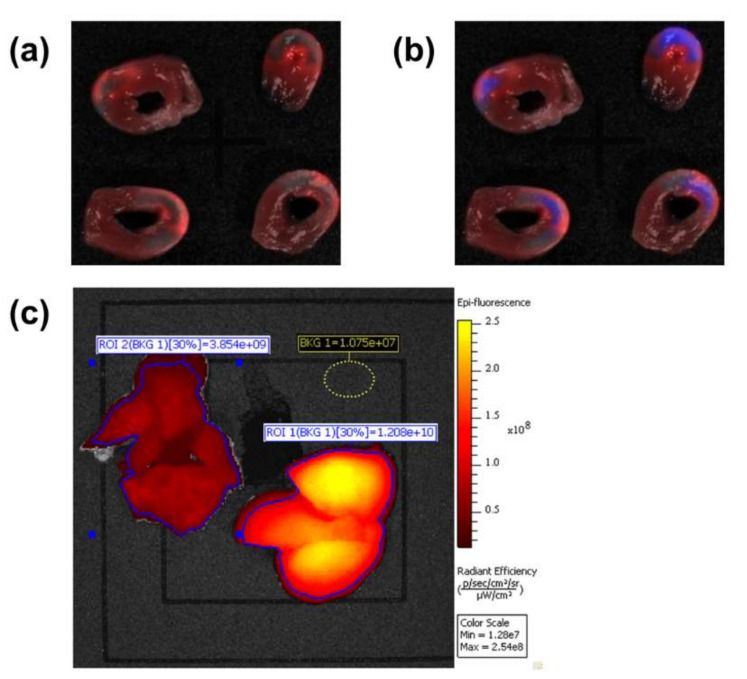
Investigation of internal organ slices harvested from laboratory animals by fluorescence tomography: (**a**) heart slices, ICG filter; (**b**) heart slices, spectral combination of filters ICG and GFP; (**c**) lung (left) and liver (right), (background, BKG subtracted).

As can be seen from Figure 12, the multilayer nanostructure accumulated in the area of the heart injury, as evidenced by the ICG fluorescence (Figure 12a). Predictably, this fluorescence does not appear in the area of non-restored blood flow. At the same time, there is a fluorescence cutoff by the GFP filter (Figure 12b), which possibly corresponds to porphyrin autofluorescence.

Conjugate accumulation was fixed in the liver (Figure 12c), which indicates its metabolic pathway through this organ. Some accumulation in the lung was also found. This phenomenon can be qualified as a side effect of accumulation in small vessels, which is characteristic of many nanoparticles.

Thus, it was proven that the multilayer nanostructure accumulates in the heart area, which indicates that a passive delivery effect occurs with this structure; therefore, it can be used in the future for the development of theranostic preparations.

## 3. Materials and Methods

### 3.1. Characteristics of the Used Nanoparticles

We used the following types of nanoparticles.

1. Commercially available silica nanoparticles (SNPs) were Aerosil ^®^ 200 from Evonik Degussa GmbH.

2. The magnetic nanoparticles were synthesized in the laboratory using the technique in [33]. To a 700 mL solution containing a mixture of iron (II) and iron (III) sulfates in a 2:1 molar ratio, a mixture of 25% ammonium hydroxide solution and 1% ammonium acetate solution was gradually added under constant stirring. The synthesis process continued until the solution started to adopt a rich black color, and the pH became equal to 8–9. The next day, the resulting nanoparticles were washed 4 times with distilled water by magnetic separation.

3. The colloidal quantum dots (CQDs) AgInS_2_/ZnS were obtained using the technique described in [34]. To 5 mL of distilled water, 0.1 mL of an aqueous solution of 0.2 M AgNO_3_, 1 mL of an aqueous solution of 0.2 M mercaptopropionic acid (MPA), and 100 μL of a 25% aqueous solution of NH_4_OH were added. The resulting solution was stirred using an anchor and a magnetic stirrer. Next, 0.1 mL of an aqueous solution of 0.4 M In(NO_3_)_3_ × 4.5 H_2_O containing 0.07 M HNO_3_ was added to the solution, and then 0.1 mL of an aqueous solution of 0.5 M Na_2_S was injected. Then, the resulting sample was heated for 30 min at a temperature of 90–95 °C. The result was a clear solution with dark red color. Then, the synthesized AgInS_2_ nuclei were covered with a ZnS shell by adding 1 mL of aqueous 0.03 M solution of MPA, 0.1 mL of aqueous solution of 0.5 M Zn(CH_3_COO)_2_, and 0.02 M HNO_3_, after which the tube containing the solution was heated for an additional 30 min.

### 3.2. Glycidine Spacer

The glycidine spacer was synthesized on the surface of SNPs and MNPs using GPMS from various solvents: cyclohexane, toluene, and benzene.

The synthesis was carried out in a 50 mL pear-shaped glass flask as follows: 2 g of nanoparticles were mixed with 23.75 mL of solvent, and then 1.25 mL of GPMS was added. The reaction was carried out in a thermostatic cell at a temperature of 80 °C for 2 h using a magnetic stirrer.

The resulting product was washed five times with cyclohexane, and then it was lyophilized at a temperature of −50 °C and an absolute pressure of 3 Pa in a VaCo 2 freeze dryer (ZirBus, Germany).

The amount of glycidine spacer was assessed by three independent methods. The first method was titration in a nonaqueous medium, as described in [35]. First, 100 mg of the sample was dispersed in 20 mL of acetic anhydride. An adequate amount of trimethylamine hydrochloride was added to the suspension. After 2 h at room temperature, the amount of released amine was determined by acidimetric nonaqueous titration with 0.1 N perchloric acid in glacial acetic acid in the presence of crystal violet indicator.

The second method was based on determining the amount of silicon in the samples. The samples were mineralized by wet ashing in nitric acid. For this procedure, each sample was boiled in 20 mL of concentrated nitric acid diluted with distilled water in a 1:1 ratio until complete dissolution in a 250 mL flat-bottomed cone-shaped flask. The cooled mineralizate was brought to 100 mL with distilled water. The mineralizate obtained in this way was analyzed for silicate content following the spectrophotometric method using the molybdenum blue method [36]. An aliquot of 2 mL was placed in a 100 mL volumetric flask, and 10 mL of a solution of iron (II) chloride at a concentration of 2 g/L and 10 mL of a solution of ammonium heptamolybdate with a concentration of 0.05 g/mL were added. The pH was adjusted to 1.3 ± 0.1 and left for 15 min. Then, 5 mL of sulfuric acid was added to neutralize the phosphate complex, and then 5 mL of reducing agents, a solution of oxalic acid with a concentration of 0.08 g/mL, and a mixture of 2.5 g of citric acid and 0.5 g of ascorbic acid per 100 mL of distilled water were added to the solution. The solution was stirred and left for 15 min to develop color and then brought to the mark with distilled water, and the relative optical density was measured on a Unico 2502S spectrophotometer (USA) at a wavelength of 830 nm in cuvettes with a layer thickness of 1 cm.

The third method was based on the amount of carbon in the sample: the sample was burned in a thin quartz tube in an oxygen atmosphere according to the Pregl method [37]. The amount of released carbon dioxide was determined volumetrically using a gas meter.

### 3.3. Immobilization of Quinacrine

Immobilization of quinacrine was carried out for 2 h at a standard temperature and at atmospheric pressure on an orbital shaker. First, 50 mg of lyophilized nanoparticles with a glycidine spacer was mixed with 2 mL of 1% quinacrine solution in anhydrous dimethyl sulfoxide (DMSO). Anhydrous DMSO was obtained by freezing at a temperature of +4 °C, with subsequent draining of unfrozen water.

The amount of immobilized quinacrine was determined spectrophotometrically. The most convenient absorption band used for quinacrine determination is the 430 nm band. The suspension obtained after chemisorption was centrifuged, and the quinacrine content in the supernatant was determined. This value was used to define the amount of the adsorbed substance.

### 3.4. Chitosan Coating

We studied SNPs and MNPs with chitosan coating obtained from a solution. As can be seen from the data presented in the analytical review on the use of chitosan as a coating of micro- or nanoparticles, chitosan coating sorption properties make it possible to use it as a spacer terminating with an amino group.

Chitosan is a biopolymer that is insoluble in water at neutral pH. It is usually dissolved in water in the presence of some organic acid and then crosslinked or converted back into the insoluble form by increasing pH [38,39].

The chitosan solution was prepared with 150 mg of chitosan dissolved in 30 mL of aqueous solution of 5% organic acid. Various acids were studied, namely, monobasic acids (acetic acid) and dibasic acids (oxalic acid, succinic acid, and tartaric acid), as well as tribasic acids (citric acid). Then, 2 mL of a chitosan solution was added to 50 mg of SNPs or MNPs; then, the substance was dispersed for 5 min in an ultrasonic disperser, and at the same time, 10% ammonia solution was added to the substance to form an opalescent colloidal solution. The amount of the added ammonia in the case of acetic, succinic, and tartaric acids was 1 mL, while in the case of citric acid, the amount was 0.2 mL. The reaction was carried out at a standard temperature and atmospheric pressure.

### 3.5. Immobilization of a Secondary Radiation Source

Using the technique in [5,6], a fluorescent dye (ICG or CQDs) was immobilized on a chitosan-based spacer grafted onto SNPs and MNPs. For this, 2 mL of ICG or CQD solution with a concentration of 0.5 mg/mL was poured onto 50 mg of nanoparticles with a chitosan-based spacer. The reaction was carried out on an orbital shaker for 2 h at standard temperature and atmospheric pressure.

### 3.6. Investigation of the Physicochemical Properties of the Samples

Micrographs of the NPs were obtained using a transmission electron microscope (TEM) with a JEM-1400 STEM field-emission cathode (JEOL, Tokyo, Japan).

The qualitative composition of the lyophilized samples was investigated using a Nicolet 8700 IR Fourier spectrometer (Thermo Scientific, Waltham, MA, USA) in bulk with the use of an integrating sphere. The spectral range of the measurements was 4000–400 cm^−1^.

The dispersed composition and average size of nanoobjects were determined using a Mastersizer 3000 laser diffraction particle size analyzer (Malvern Instruments Ltd., Malvern, Worcestershire, UK).

Nanoparticles modified with quinacrine, ICG, and CQDs were examined using an Ivis Lumina LT fluorescence tomograph (PerkinElmer, Waltham, MA, USA).

Core–shell structures were examined on a Carl Zeiss ZEN2 fluorescence microscope (Carl Zeiss AG, Oberkochen, Germany) equipped with ZEN 2012 SP2 software to obtain, process, and analyze the fluorescent images. A conjugate nanoparticle suspension sample was applied to a glass slide and dried at room temperature while preventing the ingress of dust and foreign particles.

## Figures and Tables

**Table 1:** SNP and MNP properties after GPMS chemisorption.

Sample	Specific Surface Area, m^2^/g	Concentration of Glycidine Spacer, mmol/g (μmol/m^2^)		
		Titration	Silicon	Carbon
SNP-GPMS from benzene	197	0.34 (1.7)	–	0.42 (2.2)
MNP-GPMS from benzene	70	0.27 (3.9)	0.35 (5.0)	0.30 (4.3)
MNP-GPMS from toluene	77		0.14 (1.8)	0.40 (5.2)
MNP-GPMS from cyclohexane	42		1.26 (30.0)	1.10 (26.0)

**Table 2:** Quinacrine content immobilized on a spacer obtained from different solvents.

Sample	Quinacrine Content, mmol /g
SNP-GPMS from benzene	0.007
MNP-GPMS from benzene	0.014
MNP-GPMS from toluene	0.006
MNP-GPMS from cyclohexane	0.009

**Table 3:** Measured values of luminous efficiency of fluorescent radiation of samples.

No. of Sample	Sample Composition	Total Luminous Efficiency, (p/s)/(µW/cm^2^) × 10^−8^
1	SNP-GPMS-quinacrine	99,870.0
2	MNP-GPMS-quinacrine	142.0

Note: For quinacrine 430 nm excitation filter and GFP extraction filter, the luminous efficiency value is defined by background subtraction.

**Table 4:** Chitosan coating capacitance by ICG.

No.	Acid	The Amount of ICG per Unit Mass of the Carrier, mmol/g
1	Acetic acid	0.0006
2	Succinic acid	0.0017
3	Tartaric acid	0.0026
4	Citric acid	0.0159

**Table 5:** Measured values of luminous efficiency of fluorescent radiation of samples.

No. of Sample	NP Type	Secondary Radiation Source	Acid	Total Luminous Efficiency,(p/s)/(µW/cm^2^) × 10^−8^
1	SNP	ICG	Acetic	22.8
2	SNP	ICG	Succinic	4.2
3	SNP	ICG	Tartaric	5.6
4	SNP	ICG	Citric	13.1
5	SNP	CQD	Acetic	1024
6	SNP	CQD	Succinic	1647
7	SNP	CQD	Tartaric	1524
8	SNP	CQD	Citric	931
9	MNP	ICG	Acetic	0
10	MNP	ICG	Citric	55.3
11	MNP	CQD	Acetic	23.1

Note: The luminous efficiency value is defined by background subtraction.

**Table 6:** Measured values of luminous efficiency of fluorescent radiation of the SNP-GPMS-quinacrine-chitosan-ICG (CQD) samples.

No. of Sample	Primary Shell Fluorophore	Secondary Shell Fluorophore	Light Filter for Fluorophore	Total Luminous Efficiency, (p/s)/(µW/cm^2^) × 10^−9^
1	Quinacrine	ICG	Quinacrine	9.1
	Quinacrine	ICG	ICG	0.24
2	Quinacrine	CQD	Quinacrine	300
	Quinacrine	CQD	CQD	167

Note: The luminous efficiency value is defined by background subtraction.

## Data Availability

Not applicable.
